# ZNF692 promotes cell proliferation, invasion and migration of human prostate cancer cells by targeting the EMT signaling pathway

**DOI:** 10.1186/s40001-024-01645-6

**Published:** 2024-01-30

**Authors:** Hanmin Chen, Yanmin Li, Gengqing Wu, Qingming Zeng, Haibing Huang, Guoxi Zhang

**Affiliations:** 1 Suzhou Medical College of Soochow University, Suzhou, China; 2 Department of Urology, First Affiliated Hospital of Gannan Medical University, Ganzhou, China

**Keywords:** Prostate cancer, ZNF692, Proliferation, Invasion, Migration, Apoptosis, EMT signaling pathway

## Abstract

**Background:**

Prostate cancer poses a considerable threat to human health. At present, the mechanism of tumor progression remains unclear. ZNF692 is overexpressed in many tumors, and the high expression of ZNF692 is correlated with tumor aggressiveness and tumor phenotype of prostate cancer, suggesting that ZNF692 may play an important role in tumor biology of prostate cancer. This paper aims to elucidate the relationship between them.

**Methods:**

The expression level of ZNF692 was verified in normal prostate cells (RWPE-1) and prostate cancer cells (LNCaP, PC3, DU145). PC3 cells were selected to construct the ZNF692 knockout prostate cancer cell line. The changes of cell proliferation, apoptosis, invasion and metastasis were detected by CCK8, Edu staining, Transwell assay and scratch assay. The expression levels of related proteins were detected by Western blot.

**Results:**

At the cellular level, ZNF692 was overexpressed to varying degrees in prostate cancer cell lines, with the highest expression in PC3 cell lines. CCK8 and Edu results showed that the proliferation of prostate cancer PC3 cells that knocked down ZNF692 was slowed. Transwell assay and scratch assay showed reduced invasion and migration of prostate cancer PC3 cells that knocked out ZNF692. Flow cytometry showed that the apoptosis rate of prostate cancer PC3 cells after ZNF692 knockout was increased. In addition, after ZNF692 silencing, the expression level of epithelial phenotype E-cadherin increased in PC3 cells, while the expression level of interstitial phenotype N-cadherin, Vimentin, c-Myc, and CyclinA1 decreased. The state of prostate cancer PC3 cells that overexpressed ZNF692 was reversed from the state after ZNF692 was knocked down.

**Conclusion:**

ZNF692 can be used as a new prognostic marker and a potential biologic therapeutic target for PCa. By inhibiting the expression of c-myc and cyclinA1, the EMT signaling pathway is regulated to provide evidence for its potential molecular mechanism.

## Introduction

Prostate cancer (PCa) is one of the most common malignant tumors of the male urinary system [1]. In the United States, there will be about 300,000 new cases of PCa in 2021, and 35,000 deaths from PCa. Among newly diagnosed male cancer patients, PCa accounts for 27% [2]. Although the incidence of PCa in China is significantly lower than that in the United States at present, with the improvement of living conditions, the aging of the population and the progress of medical conditions, the incidence and diagnosis rate of PCa in China have shown a significant upward trend [3]. At present, the screening of PCa mainly relies on the detection of serum prostate-specific antigen (PSA) [4]. Although PSA is highly sensitive to the detection of PCa, its specificity for PCa is poor, and its expression is affected by factors such as proliferation, inflammation and urinary retention [5]. Therefore, when an elevated PSA is found during PCa screening, a prostate biopsy is often performed for further diagnosis. Prostate biopsy is still the gold standard for PCa diagnosis [6]. Early PCa is an indolent tumor. The main treatment methods are surgery, radiation therapy and active detection. Most prostate cancers are sensitive to androgens, the androgen receptor (AR) is upregulated, producing a stimulating effect on the growth and progression of cancer cells. In addition to androgens, there is growing evidence that thyroid hormone (TH) mediates tumor promoting effects in a variety of human cancers, such as epithelial–stromal transformation (EMT), invasion and metastasis, and stimulation of angiogenesis and tumor metabolism [7, 8]. Androgen deprivation therapy (ADT) is an effective treatment and has been widely used in clinical practice [9, 10]. However, after ADT treatment, most PCa patients will progress to castration-resistant prostate cancer (CRPC), and the survival rate of patients entering this stage will be significantly reduced [11, 12]. In this study, ZNF692 avoids the influence of androgen receptors by regulating EMT-related proteins, so it is of great significance to study the mechanism of PCa occurrence and development, and search for new biologic therapeutic targets and prognostic markers.

Functional experiments and GO enrichment analysis showed that knockdown of ZNF692 inhibited the proliferation, migration and invasion of PCa cells by inhibiting the G1/S phase transition of PCa cells. Previous studies have reported that p27kip1 inhibits the cell cycle in G1 phase by inhibiting the activation of CCNE1–CDK2 complex. The distribution and prognostic value of p27kip1 in PCa tumor cells have been extensively studied [13, 14]. EMT is a process of critical importance in cancer progression that involves fundamental changes in cell phenotype that allows it to acquire the properties of interstitial cells, leading to increased aggressiveness and migration. In our model, the loss of ZNF692 was associated with altered expression patterns of EMT markers, indicating its direct role in this process.

## Methods and materials

### Cell culture

PCa cells PC3, DU145 and LNCap, and prostate hyperplasia cell BPH-1 were purchased from Institute of Biochemistry and Cell Biology, Shanghai Institute of Life Sciences, Chinese Academy of Sciences. The cell culture medium was 1640 containing 10% fetal bovine serum (FBS, Sigma-Aldrich, Cat), 100 μg/mL streptomycin and 100 μg/mL penicillin (Gibco, Cat. No. 15140-122), and they were cultured at 37 ℃ and 5% CO_2_.

### CCK-8 experiment detected the effect of ZNF692 on the proliferation of PC3 cells

PC3 cell viability was detected with CCK-8 kit (Shanghai Yanjin Biotechnology Co. Ltd, China) according to the instructions. PC3 cells cultured in 96-well plates for 24 h were taken out, and 10 μL CCK-8 reagent was added to each group of cell media and cultured in a biochemical incubator at 37 ℃ for 4 h. The absorbance (50 nm) was detected by enzyme-labeled analyzer. After calculation: cell survival rate (%) = (OD treatment group/OD control group) × 100%.

### EdU method was used to detect the effect of ZNF692 on the proliferation of PC3 cells

PC3 cells were inoculated into 6-well plates at a rate of 1.5 × 10^7^/well during logarithmic growth.The experiment was divided into three groups, control group: routine culture of inoculated cells, no administration. Low drug group: the drug concentration was 0.3 μmol L^−1^. High drug group: the drug concentration was 0.6 μmol L^−1^. After 24 h culture, according to BeyoClickTM EdU-488 cell proliferation detection kit instructions (Shanghai Biyuntian Biological Co. Ltd, China) inverted fluorescence microscope photograph. The experiment was repeated three times.

### Apoptosis experiment

2 mL of PC3 cell suspension was inoculated into each well of 6-well plate. When the cell density reached 70–80%, remove the culture medium and add salinomycin with different concentrations for 24 h. Blow and centrifuge the collected culture solution and digestive cells, and discard the supernatant. The cells were resuspended with precooled PBS, centrifuged (1000 r/min 4 ℃,10 min at 4 ℃ for 10 min), the supernatant was discarded, and this step was repeated twice. Annexin-binding Buffer (BioVision, USA, NO.1006-100) (500 μL) was added to resuspend the cells in each group, and then 5 μL of Annexin V-FITC and PI (Beijing Anolun Biotechnology Co. Ltd, China) were added, and the cells were incubated in the dark for 10 min. Three control groups were set up for each test, one group did not add any dye, and the other two groups were added with 10 μL Annexin V-FITC or PI, respectively. Apoptosis was detected by flow cytometry.

### Determination of cell migration ability

PC3 cells were inoculated in a 6-well plate and cultured overnight. When the cells fused to a monolayer, a sterile suction head with the same width was used to make a "+"scratch. PBS was used to repeatedly flush the well plate to remove cell debris, and the medium containing fetal bovine serum with a volume fraction of 0.10 was replaced. The scratch width was immediately recorded and photographed, and the cells continued to be cultured at room temperature. After 48 h, the scratch width was recorded at the same position and the healing of the scratch was compared and photographed.

### Determination of cell invasion ability

0.2 mL of PC3 cell culture supernatant was added to the lower chamber of Boyden chamber, and the upper and lower chambers were separated by a polycarbonate microporous filter membrane with an aperture of 8 μm, which was pre-coated with 50 μL of Matrigel solution (containing 120 μg of Matrigel) (Beijing Solaibao Technology Co. Ltd, China) and polymerized at 37 ℃ for 30 min to reconstruct the basement membrane. Cells treated for 96 h in each group were collected and added to the upper chamber, and 0.5 mL (containing 5 × 10^4^ cells) was added to each chamber. After 6 h incubation in a 5% CO_2_ incubator at 37 ℃, the filter membrane was taken out, and the cells that did not pass through the membrane were wiped off. After fixation, Giemsa staining was used to calculate the number of cells that passed through the membrane.

### Quantitative reverse transcription PCR (qRT-PCR)

Total RNA in cells was extracted by TRIzol reagent (Invitrogen, Carlsbad, CA). After the reverse transcription reaction, qRT-PCR was performed by using the ViiATM 7 real-time PCR system (Life Technologies, Grand Island, NY). GAPDH was the internal reference. The expression level of ZNF692 was detected by SYBR Premix Ex Taq II (Takara Biotechnology).

### Western blot

Total protein was extracted by cell lysis, that is, adding RIPA lysate (Shanghai Biyuntian Biological Co. Ltd, China), homogenizing, lysing and centrifuging to obtain total protein, then detecting the concentration of total protein by Bradford method, carrying out protein electrophoresis by SDS-PAGE (Shanghai Biyuntian Biological Co. Ltd, China), transferring the protein to PVDF membrane (Bio-Rad Corporation, US) by constant current electrotransformation, sealing the PVDF membrane in TBST sealing solution containing 5% skimmed milk powder, adding primary antibody (Shanghai Lianmai Biological Engineering Co. Ltd, China), incubating overnight at 4 ℃, washing the membrane, and adding secondary antibody (Shanghai Lianmai Biological Engineering Co. Ltd, China). Finally, the optical density of protein bands was measured by Image J analysis system software, and GAPDH protein bands were used as internal reference.

### Statistical method

Statistical software SPSS19.0 (IBM Corp., Armonk, NY, USA) was used to analyze the monitoring data. GraphPad Prism 8.0 (GraphPad Software, San Diego, CA, USA) was applied to generate graphs. The results of data analysis were all expressed as mean ± standard deviation (mean ± SD). *t* test was used for data analysis between two groups, one-way analysis of variance (ANOVA) was used for data analysis between multiple groups, and LSD test was used for subsequent analysis. *P* < 0.05 was statistically significant.

## Result

### The expression of ZNF692 in normal prostate epithelial cells and prostate cancer cells

**Fig.1:**
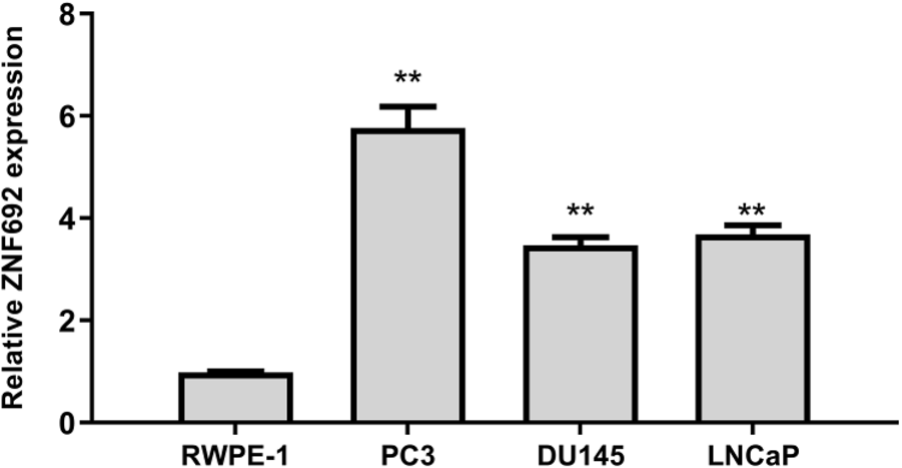
The mRNA level of ZNF692 between normal prostate epithelial cells and prostate cancer cells was evaluated by qRT-PCR. (*N* = 3). (*N* = 3). **P < 0.01

The expression of ZNF692 in normal prostate cells (RWPE-1) and three prostate cancer cells (PC3, DU145, LNCaP) was analyzed by qRCR. The results showed that ZNF692 was overexpressed in PC3, DU145 and LNCaP cell lines to varying degrees compared with RWPE-1 cell group, as shown in Fig. 1. In the follow-up experiment, PC3 was selected as the research object.

### Expression of ZNF692 in transfected cells

**Fig. 2:**
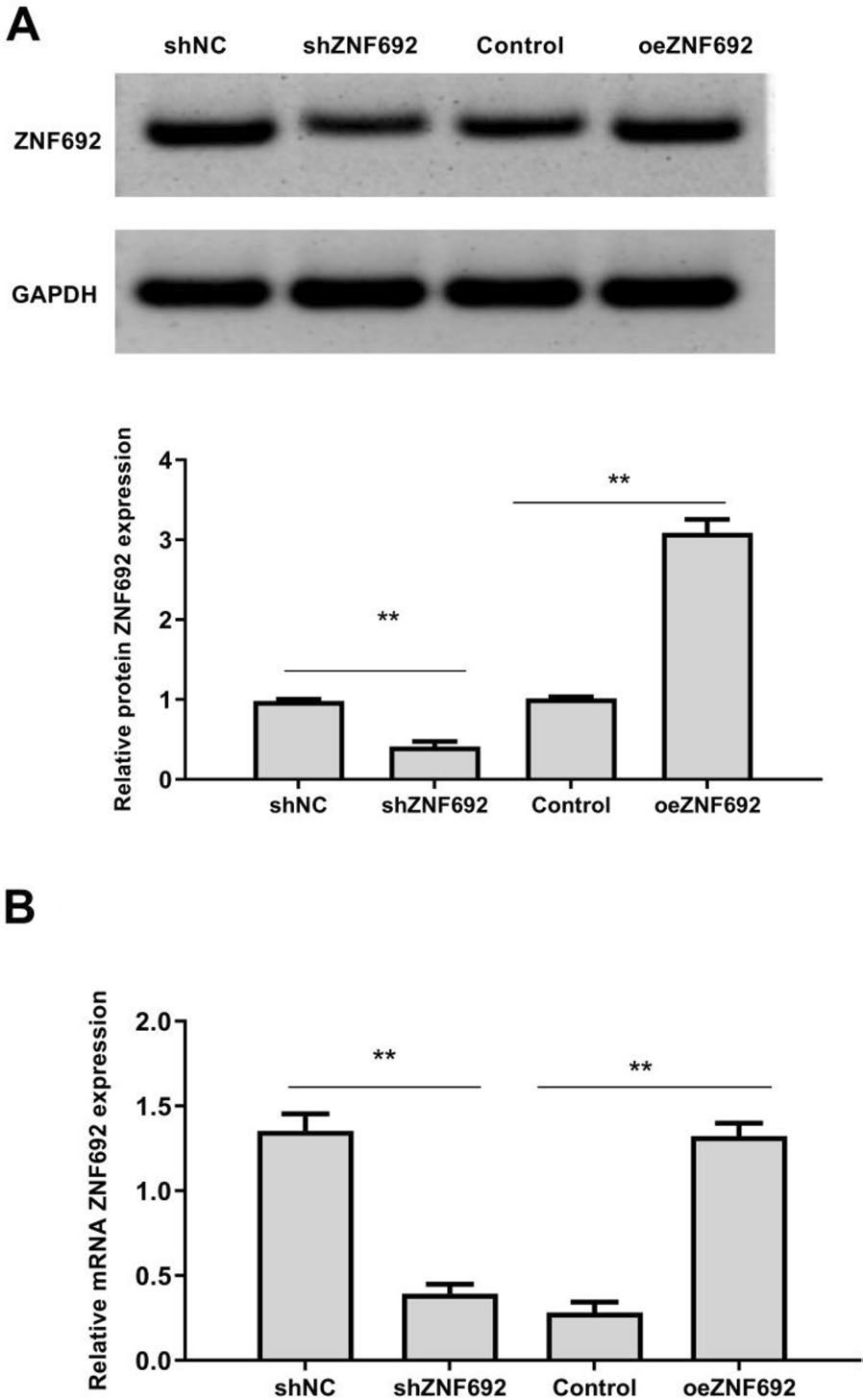
Expression of ZNF692 in transfected cells. **A** Western blot analysis of ZNF692 protein levels in transfected cells. **B** The mRNA expression of ZNF692 in transfected cells was detected by qRT-PCR (*N* = 3). ** *P* < 0.01

As shown in Fig. 2, compared with the shNC group, the expression levels of ZNF692 mRNA and protein in PC-3 cells in the shZNF692 group were significantly decreased (*P* < 0.05). Compared with the control group, the expression levels of ZNF692 mRNA and protein in PC-3 cells of oeZNF692 group were significantly increased (*P* < 0.05). The results showed that transfection was successful. We applied two sets of sequences to knock down, but we only used the efficient efficiency of silencing group.

### Effects of overexpression or interference of ZNF692 on the proliferation of PC3 cells

**Fig. 3:**
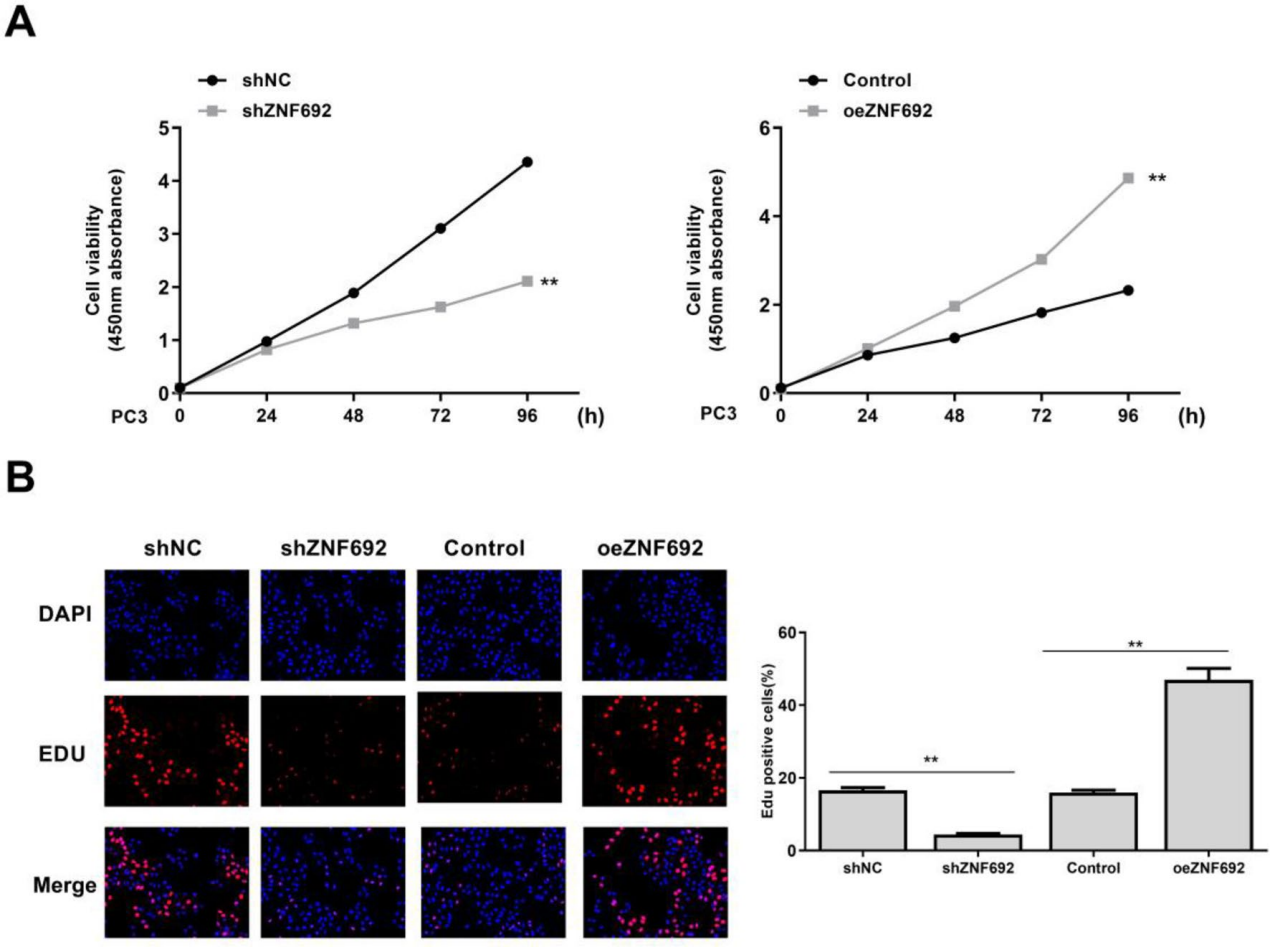
Effects of overexpression or interference of ZNF692 on the proliferation of PC3 cells. **A** CCK8 measured cell viability. **B** Edu measured cell viability (magnification, ×40; scale bar, 250 μm). (*N* = 3). **P < 0.01

As shown in Fig. 3A, the proliferation rate of PC3 cells transfected with ZNF692 overexpression vector was significantly higher than that of the control group (*P* < 0.01). The proliferation rate of PC3 cells transfected with ZNF692 interfered with shRNA was significantly lower than that of shNC group (*P* < 0.05). As shown in Fig. 3B, EdU experiment was used to verify the cell proliferation ability. The results showed that the proliferation ability of the experimental group was significantly lower than that of the control group (*P* < 0.05), and the proliferation ability of cell number after overexpression was significantly higher than that of control group (*P* < 0.05).

### Effects of overexpression or interference of ZNF692 on migration and invasion of PC3 cells

**Fig. 4:**
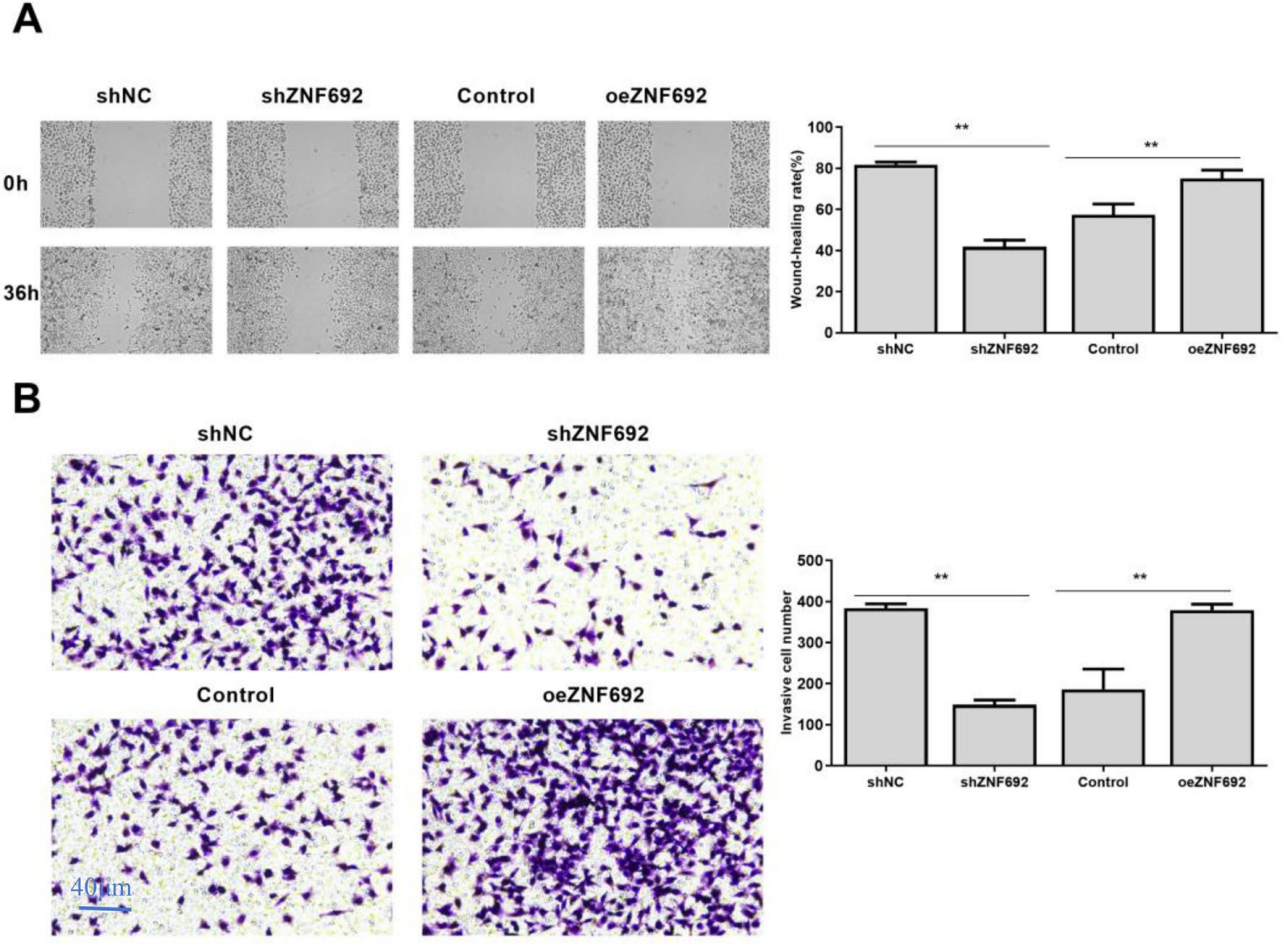
Effects of overexpression or interference of ZNF692 on migration and invasion of PC3 cells. **A** Effects of overexpression or interference of ZNF692 on migration of PC3 cells was tested by wound healing assay. **B** Effects of overexpression or interference of ZNF692 on invasion of PC3 cells was tested by Transwell assay (magnification, ×40; scale bar, 250 μm). (*N* = 3). **P < 0.01

As shown in Fig. 4A, the migration ability of PC3 cells transfected with ZNF692 overexpression vector was significantly higher than that of the control group (*P* < 0.01). After transfection with ZNF692, the migration ability of PC3 cells was significantly lower than that of shNC group (*P* < 0.05). Transwell assay was used to detect cell invasion, as shown in Fig. 4B. Transfected with ZNF692 overexpression vector, the invasion ability of PC3 cells was significantly higher than that of control group (*P* < 0.01). After transfection with ZNF692, the invasion ability of PC3 cells was significantly lower than that of shNC group (*P* < 0.05).

### Effect of overexpression or interference of ZNF692 on apoptosis of PC3 cells

**Fig. 5:**
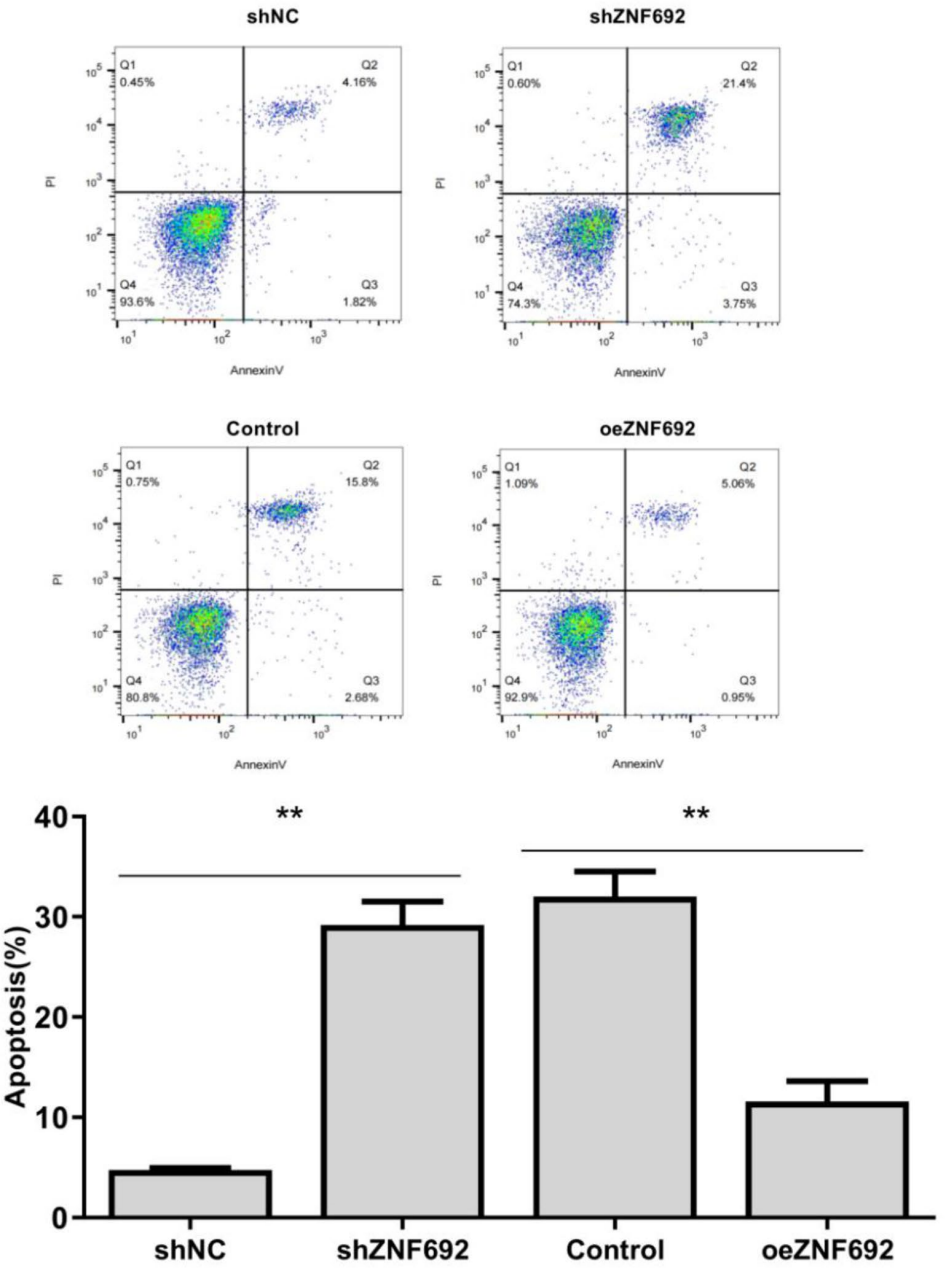
Effect of overexpression or interference of ZNF692 on apoptosis of PC3 cells was tested by flow cytometry. (*N* = 3). **P < 0.01

As shown in Fig. 5, the apoptosis rate of PC3 cells transfected with ZNF692 overexpression vector was significantly lower than that of the control group (*P* < 0.01). After transfection with ZNF692, the apoptosis rate of PC3 cells was significantly higher than that of shNC group (*P* < 0.05).

### Effect of ZNF692 overexpression or interference on EMT-related protein expression in PC3 cells

**Fig. 6:**
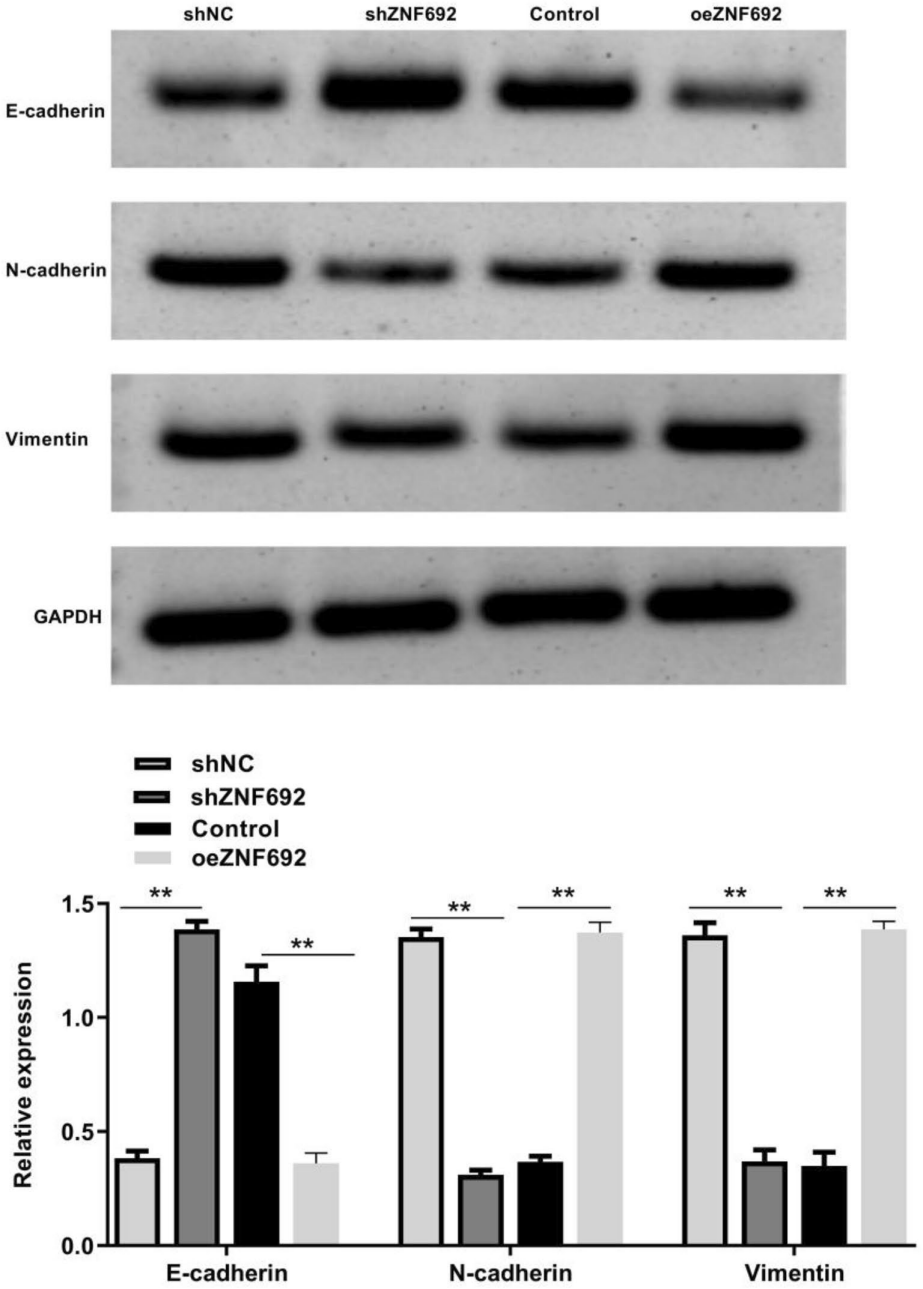
Effect of ZNF692 overexpression or interference on the expression level of EMT-related proteins in PC3 cells was detected by Western blot. (*N* = 3). **P < 0.01

As shown in Fig. 6, the expression level of the epithelial phenotype E-cadherin in PC3 cells was significantly increased after silencing ZNF692 (*P* < 0.01) While the expression levels of interstitial phenotypes N-cadherin and vimentin were significantly decreased (*P* < 0.01). It is suggested that ZNF692 silencing can inhibit the EMT process of PC3 cells. However, after transfection with ZNF692 overexpression vector, the expression of E-cadherin in PC3 cell epithelial phenotype was significantly decreased (*P* < 0.01) while the expression levels of N-cadherin and vimentin in interstitial phenotypes were significantly increased (*P* < 0.01). ZNF692 may inhibit PC3 cell invasion and metastasis by regulating EMT.

### Effects of overexpression or interference of ZNF692 on expression of c-Myc and CyclinA1 proteins in PC3 cells

**Fig. 7:**
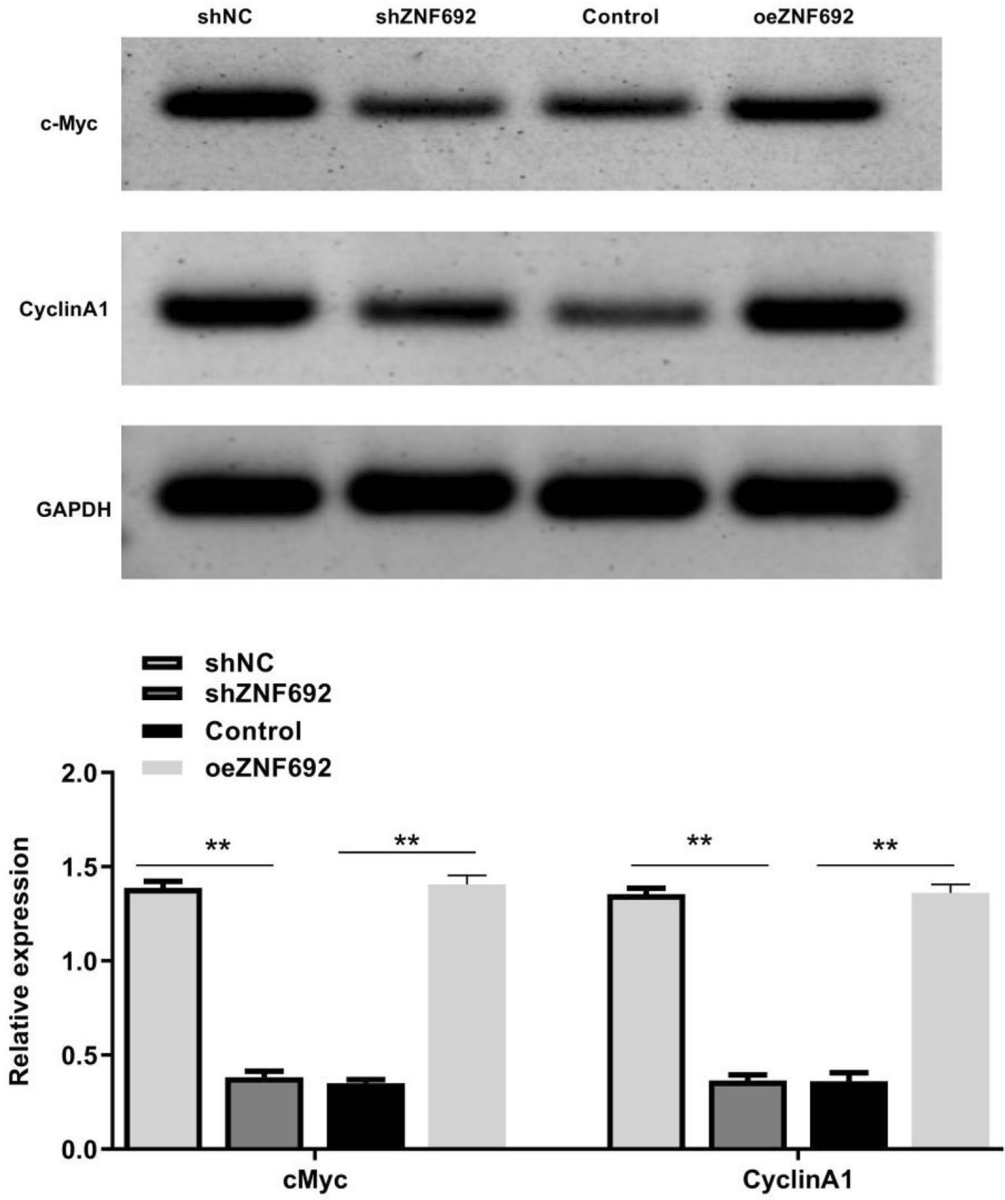
The influence of ZNF692 overexpression or interference on the expression of c-Myc and CyclinA1 proteins in PC3 cells was detected by Western blot. (*N* = 3). **P < 0.01

As shown in Fig. 7, the expression levels of c-Myc and CyclinA1 in PC3 cells were significantly reduced after ZNF692 was silenced (*P* < 0.01). However, after transfection with ZNF692 overexpression vector, the expression levels of c-Myc and CyclinA1 in PC3 cells were significantly increased (*P* < 0.01).

## Discussion

PCa is the most common malignant tumor in men. For the treatment and prognosis of PCa, the study of molecular level is particularly critical. In recent years, ZNF692 has received extensive attention as a new key molecule, and AMPK phosphorylated AREBP (ZNF692) is a key regulator of liver glucose production in vivo [15]. Overexpression of AREBP can inhibit the expression of animal gluconeogenesis gene, indicating that AREBP plays an important role in gluconeogenesis. There is increasing evidence that ZNF692 has the characteristics of an oncogene. We observed that ZNF692 is overexpressed in all PCa cell lines, especially in the highly aggressive PC3 cell line, which is an androgen-independent PCa cell that does not contain endogenous androgen receptors and avoids the influence of androgen receptors.

The role of ZNF692 in PCa appears to be multifaceted, with in vitro cell experiments showing that when it comes to cell proliferation, the loss of ZNF692 leads to a significant decrease in cell growth rate. This may be achieved through a number of pathways, including directly affecting key regulators of cell cycle progression. For example, ZNF692 is able to regulate some important cell cycle proteins, such as Cyclin A1, which are key drivers of cell division. In addition, ZNF692 may indirectly affect cell proliferation by stabilizing proteins that are harmful to the cell cycle, such as p53. Although the specific molecular mechanism remains to be further studied, the positive regulatory effect of ZNF692 on cell proliferation is clear in our experimental model. Regarding apoptosis, the knockout of ZNF692 appears to promote this programmed cell death process. Apoptosis plays a key role in maintaining tissue homeostasis, and in cancer its dysregulation is a factor leading to abnormal cell survival and tumor formation. ZNF692 may regulate apoptosis by directly or indirectly affecting the expression or function of Bcl-2 family proteins. One possible mechanism for this effect is by altering the sensitivity of cells to apoptotic stimuli, such as DNA damage or oxidative stress. In terms of cancer aggressiveness and ability to migrate, ZNF692 appears to work by influencing a process known as EMT. EMT is a process of critical importance in cancer progression that involves fundamental changes in cell phenotypes, leading to increased aggressiveness and migration. In our model, the loss of ZNF692 was associated with altered expression patterns of EMT markers, indicating its direct role in this process. Specifically, ZNF692 appears to regulate proteins associated with cell adhesion, such as E-cadherin, and factors that promote cytoskeletal modification, such as N-cadherin and Vimentin. Together, these changes promote a more aggressive cellular behavior. More broadly, ZNF692 may contribute to the development of PCa by influencing how cells interact with their microenvironment. For example, by regulating the expression of molecules on the cell surface, ZNF692 may influence cell–extracellular matrix interactions, as well as immune cell recruitment and activation. These events are critical in both local invasion and distant metastasis of the tumor. All in all, our data strongly support a central role for ZNF692 in PCa progression and provide insight into its potential as a target for future drug therapies.

EMT is a complex cell biological process that involves a fundamental change in the phenotype of epithelial cells, allowing them to acquire the properties of stromal cells. Epithelial tumor progression often involves EMT. Caterina Miro report that increased intracellular levels of hormone promote the EMT and malignant evolution of cells [16]. But ZNF692 appears to have little correlation with the hormone–hormone receptor pathway. The potential interaction between ZNF692 and EMT signaling pathway is particularly important in our study. EMT is one of the effective pathways of tumor cell migration and the most important step of tumor cell invasion and metastasis [17]. The most important feature is that the expression of E-cadherin in epithelial type is decreased, while the expression of N-cadherin and vimentin in interstitial type is increased [18]. This is called the "cadherin transition". Firstly, the regulation of the proteins E-cadherin, N-cadherin and Vimentin by ZNF692 may be achieved by directly or indirectly affecting transcription factors that play a central role in EMT. For example, ZNF692 may interact with key EMT transcription factors such as ZEB1, SLUG, or SNAIL, which directly inhibit E-cadherin transcription and activate N-cadherin and Vimentin expression. In this way, ZNF692 may be directly involved in regulating the expression of cell adhesion molecules, a key step in the EMT process. Secondly, ZNF692 may also affect multiple signaling pathways associated with EMT. For example, ZNF692 may be involved in activating pathways such as Wnt/ beta-catenin, Notch, or Hedgehog, which play a key role in the EMT process and self-renewal of cancer stem cells [19–21]. By affecting these signaling pathways, ZNF692 may not only promote EMT, but also the maintenance and proliferation of cancer stem cells, which is critical for tumor persistence and resistance to treatment. The role of ZNF692 in EMT may also involve microenvironmental influences. The tumor microenvironment, including surrounding non-cancer cells, extracellular matrix, and signaling molecules secreted by them, is known to have a profound impact on EMT and cancer progression. ZNF692 may influence the lineage of cytokines and chemokines secreted by cancer cells, thereby affecting immune cell recruitment, extracellular matrix remodeling, and the behavior of neighboring cells. In addition, ZNF692 may further promote EMT by influencing cell response to microenvironmental stimuli, such as hypoxia or inflammatory conditions. Further, ZNF692's association with cancer stem cells suggests that it may play an important role in tumor recurrence and treatment resistance. The cancer stem cell theory proposes that there is a subpopulation of cells in tumors that demonstrate enhanced self-renewal, resistance to treatment, and the ability to drive tumor recurrence and metastasis. By promoting EMT and possibly affecting cancer stem cell-related signaling pathways, ZNF692 may help maintain these cell populations with high malignant potential. However, although our study sheds light on the role of ZNF692 in EMT processes and prostate cancer progression, the detailed molecular mechanisms still require further investigation. Future studies are needed to explore in detail how ZNF692 interacts with these signaling pathways and other potentially relevant factors, and how these interactions contribute to prostate cancer aggressiveness, migration, treatment resistance, and recurrence. More extensive genomic and proteomic analyses, combined with functional experiments in vivo and in vitro, will be key to addressing these questions.

In summary, our results suggest that ZNF692 upregulates c-Myc and CyclinA1 by activating EMT signaling pathway, promotes the proliferation and resistance to apoptosis of prostate cancer cells, and enhances cell invasion and migration. These findings highlight the critical role of ZNF692 in the development and progression of PCa, indicating its importance as a potential therapeutic target.

## Data Availability

The datasets and materials used and/or analyzed during the current study are available from the corresponding author on reasonable request.If any of these declarations listed are not relevant to the content of your submission please state that this declaration is “not applicable”.
